# DNA Isolation Method Is a Source of Global DNA Methylation Variability Measured with LUMA. Experimental Analysis and a Systematic Review

**DOI:** 10.1371/journal.pone.0060750

**Published:** 2013-04-09

**Authors:** Carolina Soriano-Tárraga, Jordi Jiménez-Conde, Eva Giralt-Steinhauer, Ángel Ois, Ana Rodríguez-Campello, Elisa Cuadrado-Godia, Israel Fernández-Cadenas, Joan Montaner, Gavin Lucas, Roberto Elosua, Jaume Roquer, GeneStroke “The Spanish Stroke Genetics Consortium”

**Affiliations:** 1 Neurovascular Research Group. Neurology Department. Institut Hospital del Mar d’Investigacions Mèdiques (IMIM), Barcelona, Spain; 2 Neurovascular Research Laboratory. Institut de Recerca, Universitat Autònoma de Barcelona, Hospital Vall d’Hebron, Barcelona, Spain; 3 Laboratory of neurovascular pharmacogenomics and genetics, Fundació per la Docència i Recerca Mutua Terrassa, Terrassa (Barcelona), Spain; 4 Cardiovascular Epidemiology and Genetics group. Institut Hospital del Mar d’Investigacions Mèdiques (IMIM), Barcelona, Spain; Wayne State University, United States of America

## Abstract

In DNA methylation, methyl groups are covalently bound to CpG dinucleotides. However, the assumption that methyl groups are not lost during routine DNA extraction has not been empirically tested. To avoid nonbiological associations in DNA methylation studies, it is essential to account for potential batch effect bias in the assessment of this epigenetic mechanism. Our purpose was to determine if the DNA isolation method is an independent source of variability in methylation status. We quantified Global DNA Methylation (GDM) by luminometric methylation assay (LUMA), comparing the results from 3 different DNA isolation methods. In the controlled analysis (n = 9), GDM differed slightly for the same individual depending on extraction method. In the population analysis (n = 580) there were significant differences in GDM between the 3 DNA isolation methods (medians, 78.1%, 76.5% and 75.1%; p<0.001). A systematic review of published data from LUMA GDM studies that specify DNA extraction methods is concordant with our findings. DNA isolation method is a source of GDM variability measured with LUMA. To avoid possible bias, the method used should be reported and taken into account in future DNA methylation studies.

## Introduction

Epigenetic mechanisms regulate high-order DNA structure and gene expression without affecting the DNA nucleotide sequence. Three main epigenetic mechanisms of gene regulation have been described: DNA methylation, histone modification, and noncoding RNA.

Methylation, the most widely studied epigenetic mechanism, is a genomic DNA mark resulting from a covalent bond of a methyl group to the 5-carbon position of cytosine, generally in a 5′-CpG-3′ context. This dinucleotide is rare in the genome (∼1%) and tends to form clusters known as CpG islands, which are usually unmethylated and located in gene promoter regions. The CpG-island methylation is associated with gene silencing. However, DNA methylation also occurs at CpG island shores, in the gene body, and in repetitive elements [1]–[4]. Changes in DNA methylation contribute to inter-individual phenotypic variation and are associated with cancer development and other complex diseases [5], [6].

Global DNA Methylation (GDM) has been widely used in epidemiological studies because it is cost-effective, has a high-throughput, and provides quantitative results. GDM variation in DNA extracted from blood has been found to be associated with age, sex, alcohol consumption, and white blood cell counts [7], [8]. Global hypomethylation has also been reported in cancer cells [9]. Luminometric methylation assay (LUMA) measures levels of 5-^m^C residing in the -CCGG- motif [10], [11]. This motif, which represents 8% of all CpG sites and occurs throughout the genome [12], is used as a proxy marker to estimate global DNA methylation. However, high variability in reported GDM values makes difficult to compare different studies [7]. An unknown batch effect bias is one possible explanation for this variability.

Batch effect reflects the variability due to laboratory conditions, sample manipulation and storage, and reagent lots, where they are indistinguishable from biological results, and may lead to incorrect conclusions [13]. Collaborative studies are susceptible to batch effects because the DNA samples are measured over long periods, come from different origins, and may be handled differently.

Epigenetics is a promising field with growing interest in recent years, both because it may help in the study of complex diseases and because it may generate useful biomarkers. Reliability and consistency in GDM measurements is essential to achieving this important goal.

Previous epigenetic studies, focused on DNA methylation, have assumed that methyl groups are not lost during routine DNA extraction, but this has not been empirically tested. Classical DNA extraction consists of several steps: cell lysis, removal of lipids and proteins, and DNA precipitation. Many different methods and technologies with different protocols are available for DNA isolation. Method selection depends on several factors, such as the DNA quality and purity required and the downstream applications. Regardless of the method used, DNA samples may be exposed in varying degrees to oxidative conditions.

The aim of this study was to test whether DNA isolation method is an independent source of variability in methylation status. In this context, we also compared our results with LUMA published data, where they used different DNA isolation methods, to reinforce our hypothesis.

## Materials and Methods

### Ethics Statement

All aspects of the study were approved by the local institutional review board/institutional ethics committee for each cohort, the Clinical Research Ethics Committee of Parc de Salut Mar and the Ethics Committee of the Vall d'Hebron Hospital, Barcelona. All participants or their approved proxy provided their written informed consent for participation.

### Study Participants

We designed a 2-stages study and reviewed data of published studies based on Global DNA Methylation and its DNA extraction methods used.

#### Controlled analysis

Nine healthy donors from the Neurovascular Research Group, IMM-Hospital del Mar, were studied in 2012 [14].

#### Population analysis

We recruited 580 healthy subjects between 2005 and 2012 from three independent cohorts at the following sites in Barcelona (Spain): 359 from Cardiovascular Research Group, IMIM-Hospital del Mar (CVHM) recruited in REGICOR study; 121 from Neurovascular Research Group, Hospital Vall d’Hebron (NVVH); and 100 from Neurovascular Research Group, IMIM-Hospital del Mar (NVHM), recruited in Basicmar Register [14]. All individuals were healthy controls from each specified register.

### Demographic and Vascular Risk Factor Variables

Risk factors were collected in a structured questionnaire, as follows: arterial hypertension (evidence of at least 2 elevated blood pressure measurements, systolic >140 mm Hg or diastolic >90 mm Hg, recorded on different days before stroke onset; a physician’s diagnosis; or use of medication); diabetes (a physician’s diagnosis or use of medication); hyperlipidemia (a physician’s diagnosis, use of medication, serum cholesterol concentration >220 mg/dL, LDL cholesterol >130 mg/dL, or serum triglyceride concentration >150 mg/dl). We also recorded age, sex and current smoking habits.

### Peripheral Blood Collection. DNA Extraction Methods

DNA samples were extracted from whole peripheral blood collected in 10 mL EDTA tubes. Three different methods were used to isolate DNA (Table 1): Autopure LS (Qiagen), Puregen TM (Gentra Systems), and Chemagic Magnetic Separation Module I (Chemagen).

**Table 1:** Description and characteristics of DNA isolation methods employed in the Population Analysis (n = 580).

	Method 1 (n = 359)	Method 2 (n = 121)	Method 3 (n = 100)
*Commercial Name*	Autopure LS (Qiagen)	PuregenTM (Gentra Systems)	Chemagic Magnetic Separation Module I (Chemagen)
*System*	Automatic	Manual	Automatic
*Methodology*	Precipitation	Precipitation	Magnetic beads
*Cohort Origen*	CVHM	NVVH	NVHM

DNA concentrations were quantified using Picogreen assay and nanodrop technology. The quality of DNA samples was visualized in agarose gels.

In the controlled analysis, three 10 mL blood samples were collected from each of the 9 individuals. All blood extractions were performed at the same time and stored together at −20°C. For each individual, DNA was extracted from the blood samples using each of the three isolation methods.

In the population analysis, one 10 mL blood sample was collected from the 580 healthy individuals recruited from the 3 cohorts. DNA was extracted with a different isolation method for each cohort (Table 1).

### Luminometric Methylation Assay (LUMA)

All controlled and population GDM analyses were carried out in the same laboratory and followed a common previously described protocol, with a minor modification (see below) [11]. Genomic DNA (300 ng) was cleaved with *Hpa*II+*Eco*RI or *Msp*I+*Eco*RI (New England Biolabs) in two parallel reactions, containing 2 µl of Tango buffer (Fermentas) and 5 U of each restriction enzyme, in a final volume of 20 µl. The reactions were set up in a 96-well plate and incubated at 37°C for 4 hours. Then 20 µl of annealing buffer (20 mM Tris-acetate, 2 mM Mg-acetate pH 7.6) was added to the cleavage reactions. The original LUMA assay was modified by changing the nucleotide dispensing order to eliminate any background or nonspecific digestion of DNA samples as described previously [15]. The samples were placed in a PyroMark Q96 ID System (Qiagen) with the following dispensation order: GTGTCACAGTGT. Percentage of DNA methylation was expressed as [1 – (*Hpa*II+*EcoRI* ΣG/ΣT*)/*(*MspI+EcoRI* ΣG/ΣT)]*100. This percentage represents the amount of 5-^m^C within the CCGG motif throughout the genome.

### Statistical Analysis

We tested for association between global methylation and epidemiologic factors and the DNA isolation method used. The sample size for the population analysis was calculated on the basis of results from previous analysis (methylation results and dispersion of the variable), in order to achieve a statistical power of 90%, calculated using GRANMO v7.12. LUMA-based GDM measurements were expressed as a continuous variable and did not show normal distribution by Kolmogorov-Smirnov test. GDM was tested for univariate associations using the Kruskal-Wallis or Mann-Whitney U test for categorical predictor variables and the Spearman correlations for continuous predictor variables. In order to compare the three cohorts that constitute the “population analysis”, the predictor variables were tested for univariate associations as described above. Moreover, a multinomial regression was carried out adjusting by the variables that were significantly different at the previous univariate analysis. All statistical analyses were performed using SPSS version 18.0. A p-value of 0.05 was considered to be statistically significant.

### Review of LUMA Public Data

PRISMA guidelines for systematic reviews and meta-analyses were used [16], including checklist (Figure S1).

#### Search strategy

We reviewed the LUMA data in literature corresponding to DNA from healthy subjects. Eligible studies published before the June 2012 were identified through a Pubmed search in English. Search term combinations were as follows: “luminometric methylation assay”, “LUMA”, “global methylation” and “blood”. As exemple: luminometric methylation assay; luminometric methylation assay AND LUMA; luminometric methylation assay AND global methylation; luminometric methylation assay AND blood; luminometric methylation assay AND LUMA AND global methylation; luminometric methylation assay AND LUMA AND global methylation AND blood; LUMA AND global methylation AND blood; LUMA AND global methylation.

#### Selection criteria

Eight studies were selected on the basis of these further criteria: (i) DNA isolated from blood; (ii) Global methylation analyzed by LUMA; (iii) the specification of LUMA data for healthy subjects; (iv) the studies described the extraction DNA method, equipment, and protocols used (v) used only one DNA extraction method.

#### Data collection and quality assessment

Quantitative results were extracted from the full text article and tables. Methodological quality of included articles was assessed according to the Newcastle-Ottawa Scale (NOS) [17]. Independently, two reviewers (CST and JJC) assessed each eligible study. Disagreement was resolved by opinion of a third reviewer (EGS).

#### Data analysis

The analyses were performed using the R statistical package (version 2.11). Statistical analyses were carried out using the “rma” function of “metafor” package [18]. We applied the random effect model of DerSimonian-Laird approach to estimate the heterogeneity among studies and the I2 statistic. Moreover, meta-regression was carried out to examine the impact of DNA isolation method on study heterogeneity. The studies were grouped by DNA isolation method. The means and variance of these groups were calculated based on the means and variances weighted by the number of individuals of each study. In order to compare the mean differences, we conducted t-tests for independent samples.

## Results

### Controlled Analysis

**Figure 1:**
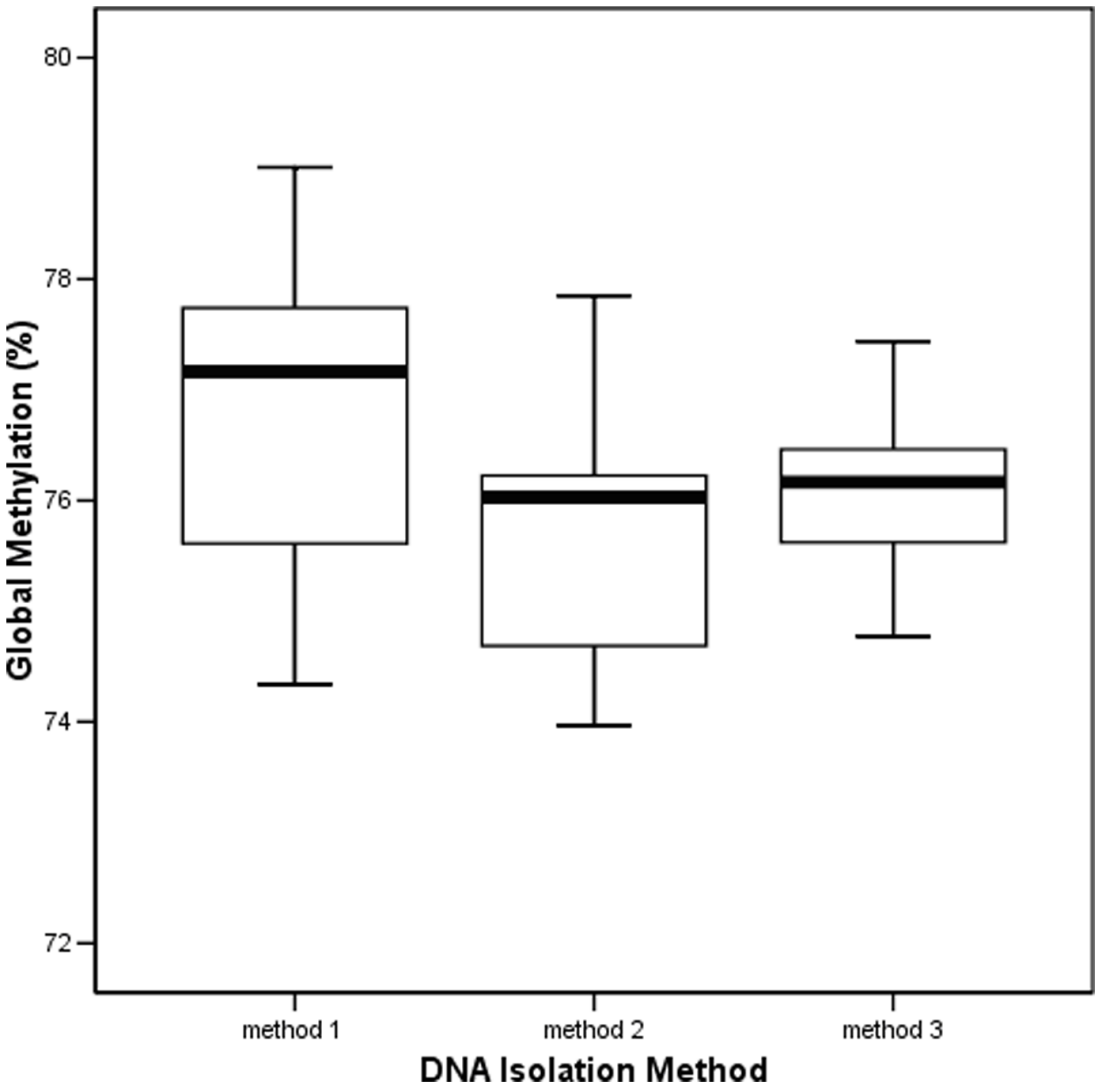
Box plot of global methylation of the 9 healthy controls using LUMA. DNA extracted by three different methods: Autopure LS (method 1), Puregen Manual kit (method 2) and Chemagic (method 3).

The mean age of participants was 28 (range 25–36), 5 were males and 2 participants were current smokers. DNA samples extracted by method 1 (Autopure LS), had a median GDM of 77.2% with an interquartile range (IQR) of 75.5–77.8; method 2 (Puregen TM), 76.0% (IQR 74.5–76.8), and method 3 (Chemagic), 76.2% (IQR 75.5–76.5). Although no statistically significant differences were found between the three methods due to the small sample size, substantial variation in values was observed, considering that all the three methods were tested on samples from the same individuals with blood extraction done at the same time (Figure 1).

### Population Analysis

**Figure 2:**
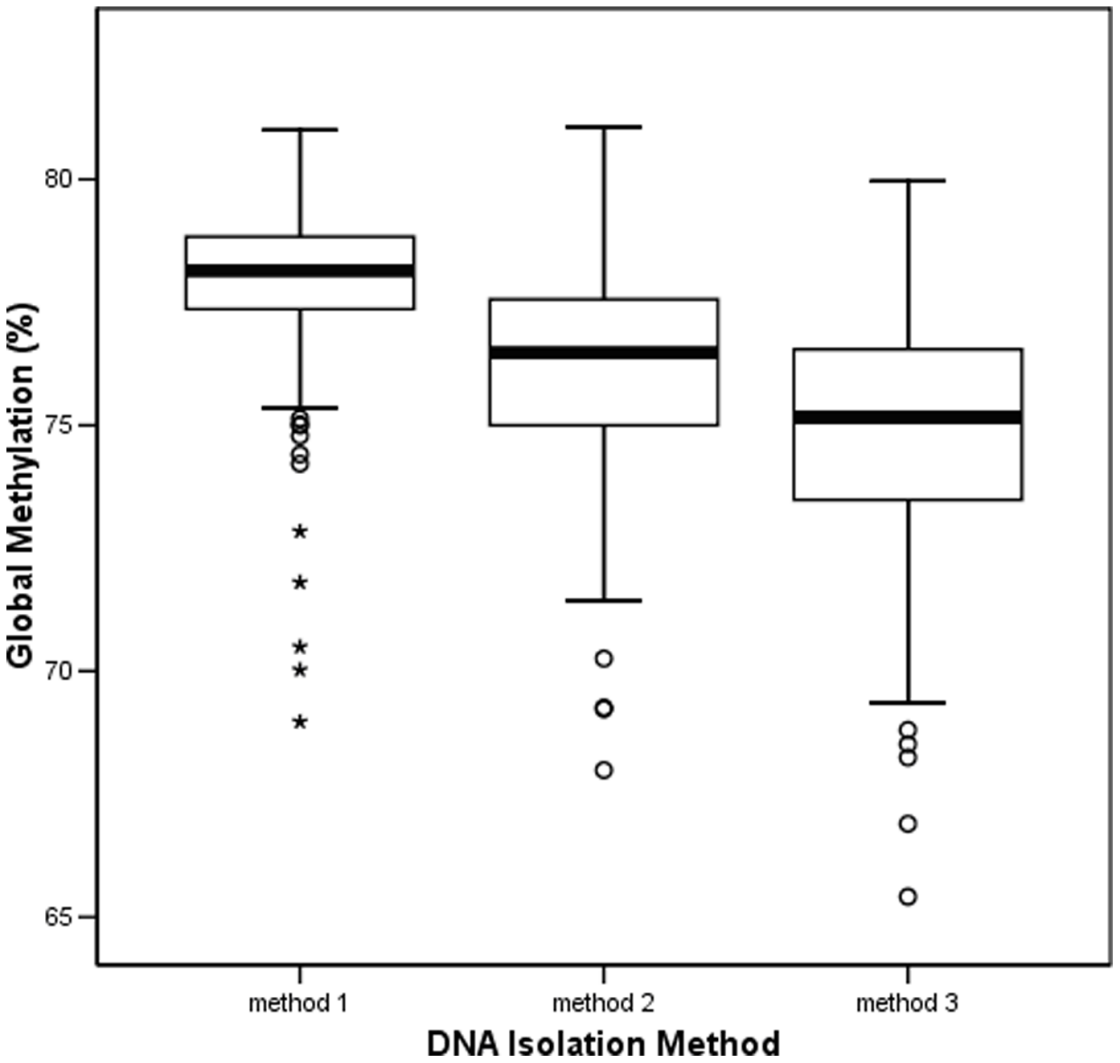
Box plot of global methylation of 580 healthy subjects using LUMA. DNA extracted by three different methods. Significant methylation differences were found between the three DNA isolation methods (medians: 78.1%, method 1; 76.5%, method 2; 75.1%, method 3; ***p<0.001).

In this stage, 580 healthy individuals were included: 359 from CVHM, 121 from NVVH, and 100 from NVHM. The clinical and demographic characteristics of the study population were as follows: mean age was 69±12.6 years, 50.2% were males (n = 291), 12.4% were current smokers (n = 72), 16.7% had diabetes (n = 97), 57.2% had hypertension (n = 332) and 38.6% had hyperlipidemia (n = 224). We observed statistically significant differences in GDM between the three DNA isolation methods, with the following median values: method 1, 78.1% (IQR 77.3–78.8); method 2, 76.5% (IQR 74.9–77.6); and method 3, 75.1% (IQR 73.5–76.6); *p*<0.001 (Figure 2). In the multivariate analysis, extraction method was the variable most significantly associated with GDM (*p*<0.001). The distribution of differences of the three isolation methods was also analyzed by Mountain plots. They can be visualized in Figure S2 that confirms these differences. Additionally, age was inversely associated with GDM (*p* = 0.024). None of the other covariables were significantly associated with GDM.

When the three cohorts that constitute the “population analysis” were compared, they showed significant differences for all the variables analyzed except for diabetes (Table 2). However, in the multivariate analysis, only the *extraction method* variable was significantly associated with GDM in all comparisons between groups (*p*<0.001).

**Table 2:** Demographic characteristics of the Population assay.

Variable	Method 1n = 359	Method 2n = 121	Method 3n = 100	P value univariate	P value multinomial regression (*/+/†)		
Age (min-max)	70 (41–84)	67 (23–89)	65 (22–88)	0.003	ns	<0.001	0.032
Gender (male %)	207 (57.7)	36 (33.6)	48 (48.5)	0.035	<0.001	ns	0.039
Smoking habit, n (%)	31 (8.8)	23 (21.5)	18 (18.9)	0.003	0.003	ns	ns
Diabetes, n (%)	68 (19)	11 (10.3)	18 (19.6)	ns	ns	ns	0.036
Hypertension, n (%)	236 (65.7)	49 (45.8)	47 (50)	0.002	ns	ns	ns
Hyperlipidimia, n (%)	165 (46.2)	28 (26.2)	31 (33.3)	0.007	0.004	ns	ns
DNA methylation (IQR)	78.1±0.7	76.5±1.35	75.1±1.5	<0.001	<0.001	<0.001	<0.001

Individuals recruited from the 3 cohorts. DNA was extracted with a different isolation method for each cohort: method 1 (Autopure) and method 2 (Gentra), method 3 (Chemagic).

* Adjusted p value for all variables in the table comparing method 1 *vs* 2 cohorts.

+ Adjusted p value for all variables in the table comparing method 1 *vs* 3 cohorts.

† Adjusted p value for all variables in the table comparing method 2 *vs* 3 cohorts.

ns, not significant.

### LUMA Public Data

**Figure 3:**
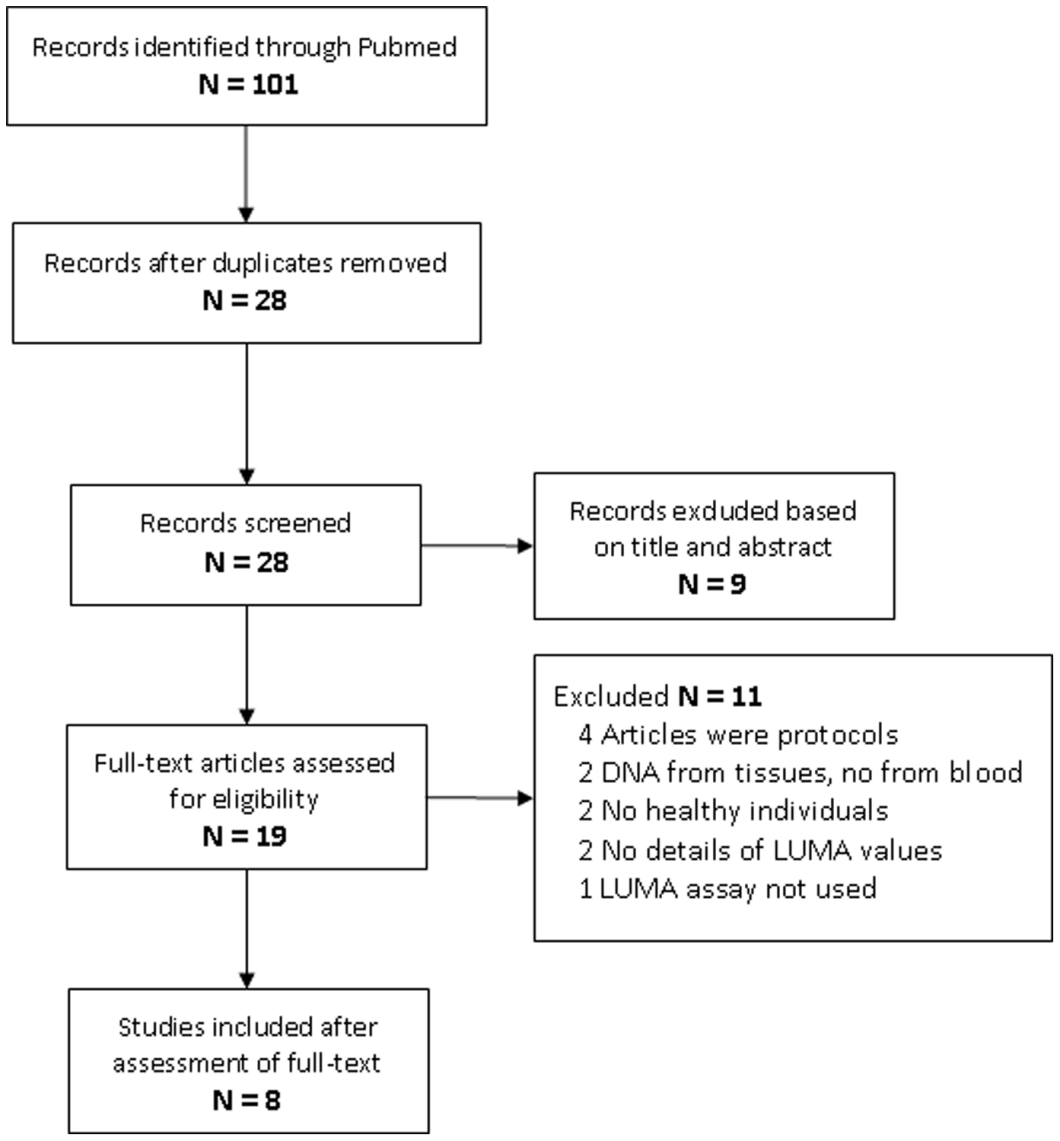
Search results on PRISMA flowchart.

**Figure 4:**
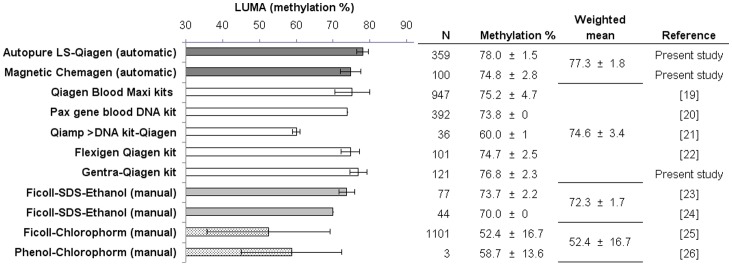
Summary of results described in the literature of global DNA methylation from blood, analyzed by LUMA. Graphic shows the methylation % of each assay, grouped by DNA isolation method: “dark grey” bars are automated method; “white” bars represent manual kits; “light grey” bars are manual Ficoll-SDS-Ethanol method and “dotted” bars are manual chloroform method. Table shows DNA isolation method employed, number of healthy subjects analyzed (N) by study, mean LUMA methylation (%) ± standard deviation (s.d.), and weighted mean ± weighted s.d. of each DNA isolation method group.

From an initial search of 101 articles identified, finally 8 were included in the systematic review (Figure 3). Quality assessment of all eight studies has been summarized in Table S1. Methylation level obtained from the DNA isolated from blood using the automated and manual methods used in the present work and by manual kits, manual Ficoll-SDS and phenol-chloroform methods of reviewed data are shown in Figure 4 [19]–[26].

**Figure 5:**
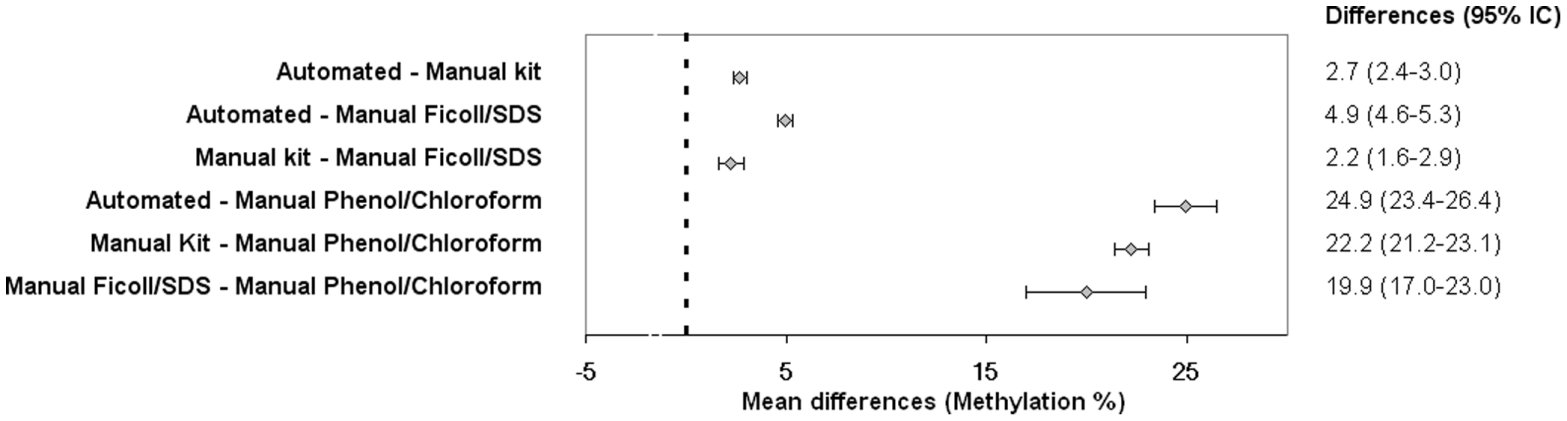
Comparison of global DNA methylation between groups of DNA isolation method. Table shows the differences of weighted means (with 95% confidence interval). All the comparisons were statistically significant (p<0.001).

The Q test for heterogeneity was highly significant between studies (p<0.0001), and the I^2^ statistic was 99%, indicating high heterogeneity. The DNA isolation method variable was significant (p = 0.02), explaining part of the heterogeneity between studies. We performed subgroup analyses by DNA isolation method. The mean methylation level of the automated DNA isolation methods was 77.3% (SD: 1.8), manual kits 74.6% (SD: 3.4), manual Ficoll-SDS 72.3% (SD: 1.7) and manual Ficoll/phenol-chloroform 52.4% (SD: 16.7). The phenol-chloroform method showed the lowest GDM (Figure 5).

## Discussion

This study demonstrates for the first time that method of DNA extraction is an important source of variability in LUMA methylation measurements. Moreover, a systematic review of previously LUMA published data of other global methylation studies confirms this variability. The differences between studies may be rather explained by the DNA extraction method batch effect.

Large epidemiological studies are susceptible to accumulate variability by differences in the protocols, sample cohorts, reagent lots, and technologies used [13]. The main problem of the batch effect is that it can be confused with biological variability. It becomes even more pronounced in collaborative studies, where different cohorts and differences in sample processing may threaten comparability of data and results [13].

DNA extraction method has not previously been taken into account as a possible source of variability in methylation studies. However, the present study demonstrates the importance of this factor. Moreover, our review of LUMA results from other studies shows differences around 20% between manual chloroform and column isolation kits, which reinforce the methylation variability described in our results. For this reason, we recommend that methylation studies that apply multiple DNA extraction methods or in cross study comparisons should adjust their methylation results by this variable.

Methylated CpG sites are frequently mutated because 5-methylcytosine (5 mC) also can be spontaneously deaminated to thymidine or oxidized by reactive oxygen species. Thus, they become rare in the genome, except at CpG islands representing less than 1% of the genome [27]. Little is known about whether DNA oxidation could result in epigenetic changes, but two studies have established an interaction between DNA methylation and oxidation [28], [29]. Cytosine and guanine of CpG sites are susceptible to oxidation. The oxidation product of 5-mC is 5-hydroxymethyluracil (HmU), and guanine oxidation results in 8-oxoguanine and both modifications can potentially interfere in the recognition of the methyl-CpG dinucleotide by methyl-CpG binding proteins.

DNA oxidation could occur during isolation by oxidants present in cells or by those produced by cell lysis [30], [31]. Some isolation methods are more susceptible to oxidation. Although there are strategies to minimize this DNA oxidation during extraction, such as omitting phenol, using antioxidants, and removing molecular oxygen [32], [33], they do not seem to fully solve the problem [30], [31]. Different levels of oxidation during the extraction procedure could decrease the methylation level. Therefore, oxidation is a possible explanation for the methylation differences between the DNA extractions methods presented in this study.

Our results also showed that age is inversely associated to DNA methylation, which is consistent with previous evidences in Alu and LINE-1 elements, revealing a global decrease in DNA methylation during aging [15], [34]. Moreover, a recent published data comparing methylomes of newborns and centenarians supports these findings [35].

A limitation of the study is that we were unable to adjust our “Population analysis” for alcohol consumption and white blood cell counts because of an excess of missing data across the three cohorts [8]. However, it is highly unlikely that these variables would completely explain such a marked association between the DNA extraction method in GDM measurement. Other limitation is that we could not take into account differences in the length of time samples were stored. To evaluate this possible source of variability we conducted a subanalysis in those cohorts for which we had the necessary information, but we did not observe any differences in the GDM measurements (data not shown). The limitations of the systematic review are the modest number studies included, the small sample size of some studies and differences in the mean age of the healthy subjects, which could explain part of the high statistical heterogeneity.

These limitations do not diminish the strengths of the study, which are based on its design and appropriate sample size. The controlled analysis, reinforced with a population analysis in 3 different cohorts and supported with data from previous studies, makes the results robust and reliable.

In summary, this study demonstrates for the first time that DNA isolation method is a source of variability in the measurement Global DNA Methylation. Isolation method should be taken into account in the design and adjustments of future DNA methylation studies.

## Supporting Information

Figure S1 **PRISMA Checklist.**
(DOC)

Figure S2 **Mountain plots.** Comparisons of the three isolation methods. A) Autopure LS and Chemagic, *versus* Gentra. B) Chemagic and Gentra *versus* Autopure LS. C) Gentra and Autopure LS, *versus* Chemagic.
(DOC)

Table S1 The Newcastle-Ottawa Scale (NOS). Assessment of the quality of studies included in the systematic review.
(DOC)
