# Supplementary figures and images for: DNA Isolation Method Is a Source of Global DNA Methylation Variability Measured with LUMA. Experimental Analysis and a Systematic Review
Source: PLoS One. 2013 Apr 9;8(4):e60750. doi: 10.1371/journal.pone.0060750 (PMC3621987; doi:10.1371/journal.pone.0060750)

**Figure S2.**

| **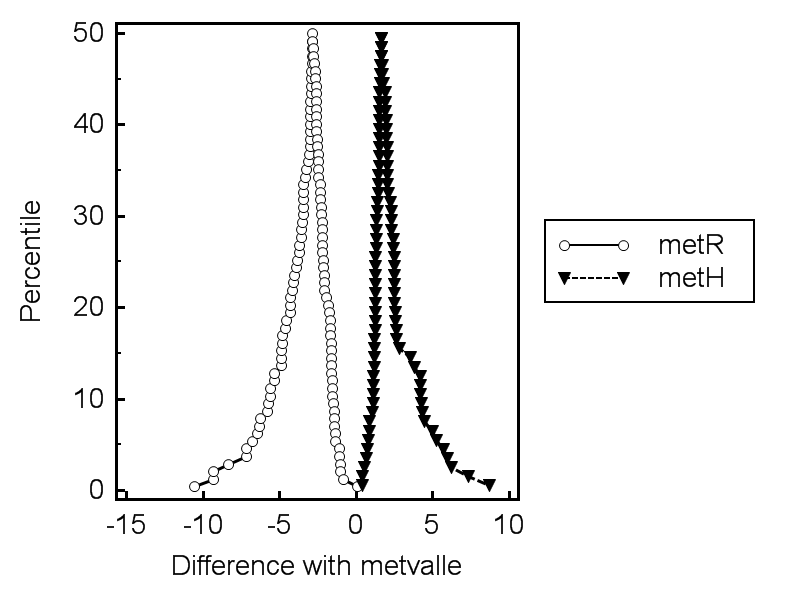**  **A** | **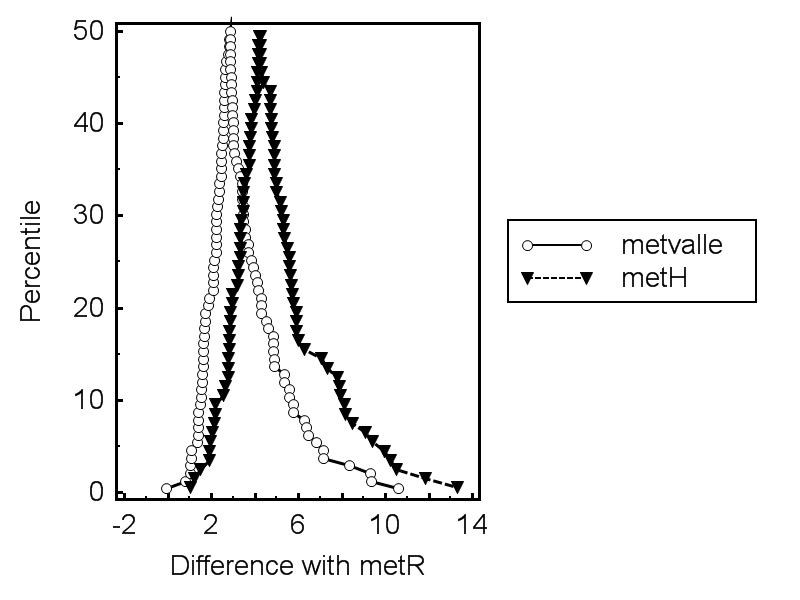**  **B** | **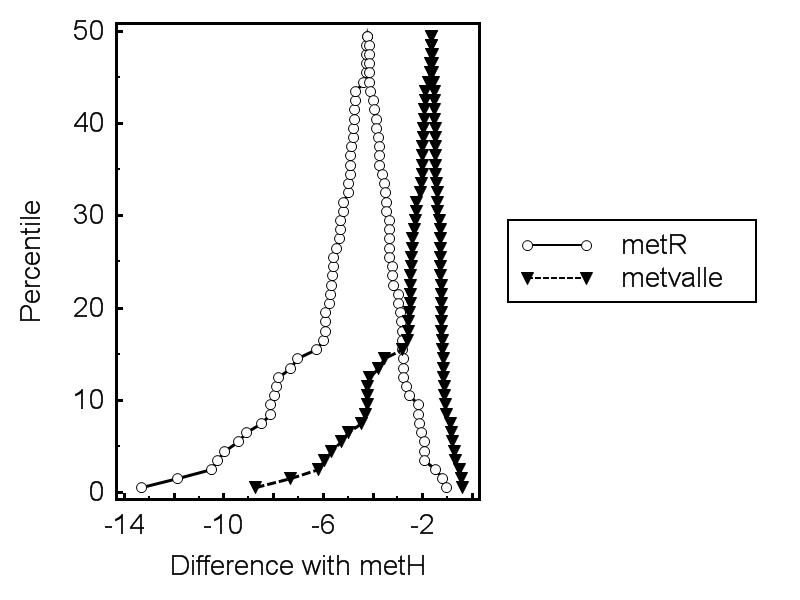**  **C** |
| --- | --- | --- |

Supplement: Figure S2 — Mountain plots. Comparisons of the three isolation methods. A) Autopure LS and Chemagic, versus Gentra. B) Chemagic and Gentra versus Autopure LS. C) Gentra and Autopure LS, versus Chemagic. (DOC) [file pone.0060750.s002.doc]
