# Supplementary material for: DNA Isolation Method Is a Source of Global DNA Methylation Variability Measured with LUMA. Experimental Analysis and a Systematic Review
Source: PLoS One. 2013 Apr 9;8(4):e60750. doi: 10.1371/journal.pone.0060750 (PMC3621987; doi:10.1371/journal.pone.0060750)
Supplement: Table S1 — The Newcastle-Ottawa Scale (NOS). Assessment of the quality of studies included in the systematic review. (DOC) [file pone.0060750.s003.doc]

**Table S1**.

| **NOS scale** | **Lumey 2012** | **Pilsner 2012** | **Virani 2012** | **Wu 2012** | **Wu 2011** | **Xu 2012** | **Sugawara 2011** | **Stenvinkel 2007** |
| --- | --- | --- | --- | --- | --- | --- | --- | --- |
| **A Selection (maximum 4)** | **3** | **2** | **4** | **1** | **3** | **4** | **2** | **3** |
| 1 Case definition adequate | 1 | 1 | 1 | 0 | 1 | 1 | 0 | 1 |
| 2 Representativeness of the cases | 1 | 1 | 1 | 0 | 1 | 1 | 0 | 1 |
| 3 Selection of controls | 1 | 0 | 1 | 1 | 0 | 1 | 1 | 1 |
| 4 Definition of controls | 0 | 0 | 1 | 0 | 1 | 1 | 1 | 0 |
| **B Comparability (maximum 2)** | **1** | **1** | **1** | **1** | **1** | **1** | **1** | **1** |
| 1 Comparability of cohorts on the basis of the design of analysis | 1 | 1 | 1 | 1 | 1 | 1 | 1 | 1 |
| **C Exposure (maximum 3)** | **3** | **2** | **2** | **3** | **2** | **2** | **2** | **2** |
| 1 Ascertainment of exposure | 2 | 1 | 1 | 2 | 1 | 1 | 1 | 1 |
| 2 same method cases and controls? | 1 | 1 | 1 | 1 | 1 | 1 | 1 | 1 |
| 3 non-response rate | 0 | 0 | 0 | 0 | 0 | 0 | 0 | 0 |
| **Total (maximum 9)** | **7** | **5** | **7** | **5** | **6** | **7** | **5** | **7** |
